# Age-Related Pathways in Cardiac Regeneration: A Role for lncRNAs?

**DOI:** 10.3389/fphys.2020.583191

**Published:** 2021-01-20

**Authors:** Francisco Santos, Magda Correia, Sandrina Nóbrega-Pereira, Bruno Bernardes de Jesus

**Affiliations:** ^1^ Department of Medical Sciences and Institute of Biomedicine - iBiMED, University of Aveiro, Aveiro, Portugal; ^2^ Faculdade de Medicina, Instituto de Medicina Molecular João Lobo Antunes, Universidade de Lisboa, Lisboa, Portugal

**Keywords:** lncRNAs, heart, regeneration, reprogramming, transdifferentiation

## Abstract

Aging imposes a barrier for tissue regeneration. In the heart, aging leads to a severe rearrangement of the cardiac structure and function and to a subsequent increased risk of heart failure. An intricate network of distinct pathways contributes to age-related alterations during healthy heart aging and account for a higher susceptibility of heart disease. Our understanding of the systemic aging process has already led to the design of anti-aging strategies or to the adoption of protective interventions. Nevertheless, our understanding of the molecular determinants operating during cardiac aging or repair remains limited. Here, we will summarize the molecular and physiological alterations that occur during aging of the heart, highlighting the potential role for long non-coding RNAs (lncRNAs) as novel and valuable targets in cardiac regeneration/repair.

## Introduction

Worldwide, cardiovascular diseases are the leading cause of death, causing nearly 18 million deaths in 2017. Cardiovascular diseases comprise several pathological conditions, including heart failure (Yusuf et al., 2001; Lloyd-Jones et al., 2009, 2010; Mensah et al., 2019). Aging is probably the most important risk factor for heart failure (Li et al., 2020a). As opposed to the neonatal heart, adult mammalian hearts lose their capacity to fully regenerate after an exogenous or endogenous harm (Lam and Sadek, 2018). This may be mediated through several interconnected processes, including cellular senescence and secreted factors, telomere attrition, mitochondrial damage, cell death, or inflammation (for a comprehensive review on age-related pathways affecting the heart, see Li et al., 2020a). Although a partial myocyte turnover has been observed in adult heart after damage (e.g., myocardial infarction), it only partially and slightly restores heart function. For instance, it has been recently demonstrated that manipulation of telomere length through the expression of telomerase, whose expression is silenced in the mouse heart from day 5 to 7 (Blasco et al., 1995; Borges and Liew, 1997; Richardson et al., 2012), may be beneficial in heart healing and healthspan (Bernardes de Jesus and Blasco, 2011; Bar et al., 2014).

Long non-coding RNAs (lncRNAs) have emerged as important regulators of epigenetic modulation and gene expression. Although deprived from coding potential, lncRNAs have been associated with several biological processes, including dosage compensation, genomic imprinting, aging, and cell differentiation (Mercer et al., 2009; Rinn and Chang, 2012; Sousa-Franco et al., 2019; Yao et al., 2019). Furthermore, lncRNAs have been linked to several diseases including cardiovascular diseases (Hobuß et al., 2019; Abbas et al., 2020). In this review, we will discuss the cardiac regeneration properties of neonatal and adult hearts. We will focus on lncRNAs and their potential role in cardiac regeneration briefly discussing the potential of fibroblasts as a source of cardiomyocytes for regenerative medicine purposes.

## Cardiac Regeneration in Neonatal and Adult Hearts

There is a general consensus on the capacity of neonatal hearts to regenerate, after distinct types of damage (Supplementary Figure 1 – Porrello et al., 2011, 2013; Haubner et al., 2012, 2016; Jesty et al., 2012; Mahmoud et al., 2013, 2014, 2015; Rubin et al., 2013; Andersen et al., 2014, 2016; Aurora et al., 2014; Sadek et al., 2014; Bryant et al., 2015; Darehzereshki et al., 2015; Han et al., 2015; Jiang et al., 2015; Konfino et al., 2015; Aix et al., 2016; Blom et al., 2016; Kang et al., 2016; Tao et al., 2016; Valiente-Alandi et al., 2016; Xiong and Hou, 2016; Yu et al., 2016; Ai et al., 2017; Bassat et al., 2017; Malek Mohammadi et al., 2017; Zebrowski et al., 2017; Ahmed et al., 2018; Ingason et al., 2018; Sampaio-Pinto et al., 2018; Sereti et al., 2018; Cai et al., 2019; Elhelaly et al., 2019; Wang et al., 2019b; Fan et al., 2020; Pei et al., 2020; Li et al., 2020b, 2020c). A comprehensive overview of neonatal heart regeneration studies has been previously and elegantly detailed by Lam and Sadek (2018). Heart regeneration seems to be dependent on the type of injury that causes loss of cardiomyocytes. For example, cryoinjury does not induce the same level of regeneration as apical resection or myocardial infarction. Furthermore, it is likely that neonatal heart regeneration is mediated by the proliferation of pre-existing cardiomyocytes, and not by cardiac stem or progenitor cells. This regenerative state occurs in an extremely short time frame (<10days; Eschenhagen et al., 2017) and, just a few days after birth, cardiomyocytes exit the cell cycle resulting in a decline in heart regeneration capacity. This is accompanied by other alterations in the cardiomyocytes, including their metabolic needs or changes in the expression of both coding and non-coding genes. Subsequently, several strategies have been designed for regenerating the adult heart. Those approaches may include the forced re-entering in the cell cycle of the pre-existing cardiomyocytes, or may include cell transdifferentiation strategies, in which somatic cells can be converted into functional cardiomyocytes for cell replacement therapy (Qian et al., 2012; Addis and Epstein, 2013; Nam et al., 2013; Wada et al., 2013; Ghiroldi et al., 2017; Amin et al., 2018; Engel and Ardehali, 2018a).

## A Role for Lncrnas in Heart Regeneration

The importance of lncRNAs in heart regeneration has been brought to light recently (Bar et al., 2016; Abbas et al., 2020). LncRNAs are a vast category of non-coding, poorly conserved, and tissue- and developmental stage-specific transcripts with distinct functions in several biological processes, including epigenetic, transcriptional, and post-transcriptional regulation. Regarding the role of lncRNAs in heart regeneration, we will discuss some recent studies describing lncRNAs directly acting (promoting or inhibiting) on heart regeneration (Table 1).

**Table 1:** LncRNAs with reported roles in cardiac regeneration.

LncRNA	Reported role in cardiac regeneration	Reference
Negative regulators		
AZIN2-sv	↓ cardiomyocyte proliferation by sequestering miR-214 and leading to a decrease in the phosphorylation of Akt and Cyclin D	Li et al., 2018b
CAREL	↓ cardiomyocyte proliferation by sequestering miR-296 and activating Trp53inp1 and Itm2a	Cai et al., 2018
CPR	↓ cardiomyocyte proliferation by the recruitment of DNMT3A, leading to increased levels of methylation of the MCM3 promoter	Ponnusamy et al., 2019
CRRL	↓ cardiomyocyte proliferation by sequestering miR-199a-3p, leading to an increased expression of Hopx	Chen et al., 2018
LncDACH1	↓ cardiomyocyte proliferation by regulating PP1A/YAP1 signaling	Cai et al., 2020a
SARRAH	↑ cardiomyocyte apoptosis by increasing caspase activity	Trembinski et al., 2020
Positive regulators		
NR_045363	↑ cardiomyocyte proliferation *via* the miR-216a/JAK-STAT3 pathway	Wang et al., 2019a; Chen et al., 2020
	↓ cardiomyocyte apoptosis by blocking p53 activation	
ECRAR	↑ cardiomyocyte proliferation by promoting phosphorylation of ERK1/2 to activate Cyclins D1 and E1	Chen et al., 2019
Sirt1 antisense lncRNA	↑ cardiomyocyte proliferation and ↓ cardiomyocyte apoptosis by stabilizing Sirt1	Li et al., 2018a

### LncRNAs That Promote Cardiomyocyte Proliferation and Cardiac Regeneration

P7 mice subjected to LAD ligation and injected with adenovirus containing NR_045363 exhibited improved left ventricular ejection fraction and reduced infarct size compared to the control-injected group (Wang et al., 2019a). Mice overexpressing NR_045363 showed higher expression of cardiomyocyte mitotic markers, such as Ki67 and phosphorylated histone H3 (pH3), suggesting that improved heart function after MI was due to cardiomyocyte proliferation. The authors reported that NR_045363 acted as a competing endogenous RNA (ceRNA), binding to miR-216a (Wang et al., 2019a). miR-216a is known to repress JAK2, leading to decreased levels of phosphorylation of STAT3 (Hou et al., 2015). Furthermore, deletion of STAT3 was shown to impair cardiomyocyte proliferation after apical resection (Kurdi et al., 2018), suggesting that NR_045363 promoted cardiomyocyte proliferation by modulating the JAK2-STAT3 pathway. So, the absence of NR_045363 (which results in an upregulation of miR216a) led to reduced activity of the JAK2-STAT3, whilst NR_045363 overexpression (which leads to a downregulation of miR-216a) resulted in an increase of the phosphorylation levels of JAK2 and STAT3, thus promoting cardiomyocyte proliferation (Wang et al., 2019a). More recently, NR_045363 was associated with cardiomyocyte apoptosis. Chen et al. (2020) reported that loss of NR_045363 led to the activation of the p53 signaling pathway, promoting apoptosis. On the other hand, overexpressing NR_045363 inhibited apoptosis and improved cardiac function after MI, thus potentially mediating the cardiac functions observed after NR_045363 modulation.

Long non-coding RNA endogenous cardiac regeneration-associated regulator (ECRAR) was found to be upregulated in the fetal heart, and its expression gradually decreased in postnatal hearts. Overexpression of ECRAR in postnatal rat cardiomyocytes, both *in vitro* and *in vivo*, resulted in an increase of DNA synthesis, and an increase of cytokinesis (pH3 and aurora B kinase), suggesting a direct involvement in cardiomyocytes proliferation (Chen et al., 2019). Overexpression of ECRAR resulted in the phosphorylation of ERK1/2, their subsequent translocation to the nucleus and the transcription of cell proliferation and cell cycle-related genes (Chen et al., 2019). Li et al. (2018a) identified Sirt1 antisense lncRNA (Sirt1-as), whose expression was high during heart development. Overexpression of this lncRNA resulted in an increase of Ki67- and pH3-positive cardiomyocytes. On the other hand, silencing of Sirt1-as, both *in vitro* and *in vivo*, led to a decrease of Ki67- and pH3-positive cardiomyocytes, indicating a potential decline in cell division (Li et al., 2018a). Furthermore, overexpression of Sirt1-as after MI in adult mice resulted in an increased expression of cell-cycle specific factors Ki67 and pH3, thus suggesting a potential implication in cardiac health (Li et al., 2018a).

More recently, Wilson et al. (2020) described BANCR, a lncRNA exclusively expressed in primate fetal cardiomyocytes. BANCR promotes cardiomyocyte migration *in vitro* and ventricular enlargement *in vivo*. To elucidate the regulation of BANCR in cardiomyocytes, the authors suggested that TBX5 binding was responsible for the fetal heart-specific expression of BANCR. Additionally, the authors identified TEAD4 and YAP1 (two factors involved in the HIPPO pathway) in the same enhancer, promoting BANCR expression. Finally, the authors identified a role for BANCR in heart disease, demonstrating higher expression in pediatric but not adult dilated cardiomyopathy (Nelakanti and Xiao, 2020; Wilson et al., 2020). Other lncRNAs associated with aged hearts include lncRNA H19 (downregulated in aged or ischemic heart; Hofmann et al., 2019), and MALAT1 a lncRNA which, itself, is regulated by an antisense lncRNA transcript (TALAM1; Zong et al., 2016; Gomes et al., 2019), was also shown to be decreased in aged hearts (Bink et al., 2019; Gomes et al., 2019), and this decrease was shown to be involved in cardiac dysfunction (Zhu et al., 2019; Li et al., 2020a).

### LncRNAs That Inhibit Cardiomyocyte Proliferation and Cardiac Regeneration

Cai et al. (2018) explored the role of lncRNAs during heart regeneration after ischemic injury, in both neonatal and adult mice. *CAREL*, a lncRNA whose expression gradually increased in the neonatal hearts from P1 to P10 mice, with P7 corresponding to the time point at which the heart regenerative capacity is lost in mice (Cai et al., 2018). Cardiac-specific overexpression of *CAREL* led to a decrease of cardiomyocyte proliferation and reduced heart regeneration in neonatal mice after injury. On the contrary, silencing *CAREL* promoted cardiac regeneration and improved heart functional parameters after myocardial infarction in neonatal and adult mice (Cai et al., 2018). *CAREL* was found to be a ceRNA, sequestering miR-296. It was suggested that the *CAREL-miR-296* interaction led to the activation of Trp53inp1 and Itm2a, leading to a decrease in cardiomyocyte proliferation, thus resulting in a reduction of regeneration. Intramyocardial administration of *CAREL* to p1 neonatal mice inhibited cardiomyocyte mitosis and increased the formation of cardiac scar and, on the other hand, overexpression of miR-256 promoted cardiomyocyte proliferation and cardiac regeneration after injury. Similarly, lncRNA cardiomyocyte proliferation regulator (CPR) was shown to be a negative regulator of cardiomyocyte proliferation and cardiac repair. Ponnusamy et al. (2019) observed that higher levels of CPR hampered cardiomyocyte proliferation, whilst silencing CPR resulted in cardiomyocyte proliferation in postnatal and adult hearts. CPR expression levels were found to be higher in the adult heart, which is consistent with their lack of regeneration. The authors reported that CPR recruits DNMT3A to several locus leading, in particular, to increased levels of methylation in the MCM3 promoter (Ponnusamy et al., 2019). In dividing tissues, MCM3 promotes the initiation of DNA replication and cell cycle progression (Lin et al., 2008), something halted by CPR in the heart and leading to the inhibition of cardiomyocytes proliferation.

Another lncRNA that negatively regulates cardiac regeneration is LncDACH1. This lncRNA was found to be gradually upregulated in postnatal hearts, which is in accordance with the loss of myocardial regenerative capacity soon after birth (Cai et al., 2020). The authors suggest that LncDACH1 binds protein phosphatase 1 catalytic subunit alpha (PP1A), reducing its dephosphorylation capacity, and increases the phosphorylation of yes-associated protein 1 (YAP1), preventing its translocation to the nucleus and, thus, the activation of cell proliferation-related genes (von Gise et al., 2012; Cai et al., 2020). Cardiac-specific overexpression of LncDACH1 resulted in the suppression of neonatal heart regeneration and aggravation of cardiac function after apical resection. These phenotypes were accompanied by a decrease in the number of cardiac-cells expressing proliferative markers (Cai et al., 2020). Cardiomyocyte regeneration-related lncRNA (CRRL) was also found to be involved in heart regeneration. CRRL silencing was associated with an increased expression of EdU, Ki67, and pH3 in P1 and P7 rat cardiomyocytes (Chen et al., 2018). Similar results were obtained in neonatal rats post-MI, concomitantly with better prognosis such as reduction of the fibrotic length of the infarct wall and fibrosis area in the non-infarct zone. Instead, overexpression of CRRL leads to a decrease in pH3-positive cardiomyocytes and inhibition of functional recovery post-MI. CRRL function seemed to be mediated through the binding to miR-199a-3p, resulting in an increased expression of *Hopx*, which is a negative regulator of cardiomyocyte proliferation (Trivedi et al., 2010).

LncRNA AZIN2-sv, a splice variant of the AZIN2 gene, was found to be upregulated in human adult hearts. AZIN2-sv was reported to negatively regulate cardiomyocyte proliferation, both *in vitro* and *in vivo* (Li et al., 2018b). Overexpression of AZIN2-sv led to an anti-proliferative phenotype, marked by decreased levels of EdU-, Ki67-, pH3-, and Aurora-B. On the other hand, silencing AZIN2-sv promoted cardiomyocyte proliferation and improved cardiac function after MI. AZIN2-sv sequesters miR-214, leading to the release of its target PTEN, resulting in a decrease in the phosphorylation of Akt and Cyclin-D, therefore inhibiting cardiomyocyte proliferation. Reduced levels of AZIN2-sv allow miR-214 to repress PTEN, leading to increased levels of phosphorylated Akt and Cyclin-D1, thus promoting cardiomyocyte proliferation.

More recently, Trembinski et al. (2020) identified lncRNA SCOT1-antisense RNA regulated during aging in the heart (*SARRAH*), whose expression declines during aging. Inhibition of *Sarrah* induces caspase activity in mouse and human cardiomyocytes, promoting apoptosis. Gene set enrichment analysis after *SARRAH* silencing showed enrichment of apoptosis-related pathways, corroborating previous observations (Trembinski et al., 2020). *SARRAH* was also found to directly bind to the promoters through RNA-DNA triplex helix structures, suggesting that its binding may activate gene expression. Indeed, it was reported that *SARRAH* interacted with cardiac transcription factor cysteine-rich protein 2 (CRIP2) and p300, which acetylates histone H3 lysine 27 to activate transcription (Trembinski et al., 2020). On the other hand, overexpression of SARRAH led to a decrease in caspase activity. In adult mice a decline in apoptosis was observed after overexpressing SARRAH, suggesting that reduced expression levels of this lncRNA in aged mice might contribute to cardiomyocyte cell death *in vivo*. Furthermore, reduced levels of Sarrah were observed in the infarcted and border regions after acute MI (Trembinski et al., 2020).

Furthermore, several lncRNAs have been identified as promoters of cardiac fibrosis (Liang et al., 2018; Wang et al., 2018; Hao et al., 2019; Zhang et al., 2019a). Aged tissues accumulate signals that promote the epithelial-mesenchymal transition (EMT), inducing the transdifferentiation of epithelial cells to mesenchymal cells, such as fibroblasts, which are the main mediators of fibrosis through the deposition of extracellular matrix. In the heart, many fibroblasts derive from endothelial cells, leading to excessive deposition of extracellular matrix and causing cardiac fibrosis, which is common in patients with heart failure (reviewed by Santos et al., 2019). Thus, targeting these lncRNAs may also be considered for improving heart function.

## Reprogramming of Fibroblasts Into Cardiomyocytes as a Potential Cell Replacement Therapy – a Role For Lncrna

Cell reprogramming has emerged as a novel strategy for regenerative medicine and cell-based therapy. The reprogramming of mouse and human fibroblasts into induced pluripotent stem cells (iPSCs) using transcription factors (TFs) known to play key roles in the maintenance of embryonic stem cell identity suggested that patient-derived iPSCs could be produced from somatic cells. This strategy allowed the conversion of fully differentiated cells into cells with the potency to be differentiated in tissues from different development lineages (Takahashi and Yamanaka, 2006; Takahashi et al., 2007; Yamanaka, 2009; Abad et al., 2013). Additionally, many of the reprogramming barriers, such as the obstacles imposed by aging, have been addressed through the direct manipulation of tumor suppressor genes, p53 or Ink4a/ARF (Li et al., 2009; Marion et al., 2009), or the EMT-promoting factor ZEB2 (Bernardes de Jesus et al., 2018; Santos et al., 2019).

The reprogramming of fibroblasts into iPSCs opened doors to direct cell reprogramming. Direct cardiac reprogramming of fibroblasts into cardiomyocytes [usually termed induced cardiomyocytes (iCMs)] has emerged as an attractive strategy for replacing lost or damaged cells in the heart. Mouse postnatal cardiac and dermal fibroblasts have been transdifferentiated into iCMs through the combined expression of three different cardiac-specific TFs: *Gata4*, *Mef2c,* and *Tbx5* (GMT). The ectopic expression of GMT activates a cardiac-like gene expression program and promotes the conversion of fibroblasts into iCMs (Ieda et al., 2010; Qian et al., 2012). Comparative gene expression analyses reported that iCMs generated *in vitro* exhibited *bona fide* adult cardiomyocyte-like features, such as fatty acid oxidation or cell cycle exit (Muraoka et al., 2019). Remarkably, this approach has been adapted *in vivo*, where cardiac fibroblasts have been transdifferentiated into iCMs (Song et al., 2012; Zhang et al., 2019b, 2019c), bypassing the need to revert fibroblasts to a pluripotent state (Liu et al., 2017; Muraoka et al., 2019). Endogenous cardiac fibroblasts comprise about 50% of all the cells in the heart, making them a potential source of cardiomyocytes for regenerative therapy (Ieda et al., 2010). In fact, iCMs reprogrammed from endogenous cardiac fibroblasts enhanced cardiac function after myocardial infarction, fully demonstrating the potential of this strategy for cardiac repair (Miyamoto et al., 2018; Bektik and Fu, 2019; Lee et al., 2020).

Despite several encouraging results, current reprogramming methodologies remain somewhat inefficient, as very few fibroblasts are fully converted into functional iCMs. Differential expression patterns of lncRNAs have been observed in several developmental stages, including cardiogenesis, and involve the expression of lncRNAs *Braveheart*, *Fendrr,* and *Carmen*. In fact, lncRNA *ZEB2-NAT* has been modulated in order to improve the reprogramming of fibroblasts into iPSCs (Bernardes de Jesus et al., 2018). Having these concepts in mind, it seems reasonable to expect that modulating lncRNAs might improve the efficiency of direct cardiac reprogramming.

### *In vivo* Therapeutic Delivery – Current Issues

Regarding phenoconversion of cardiac cells, it is important to mention, however, that many of the current protocols depend on viral vectors for gene delivery. There are a few safety issues associated with the use of lentiviral and retroviral vectors, as they integrate their genome in the host cell. They could potentially disturb endogenous gene expression and are associated with the risk of insertional mutagenesis, hampering the clinical application of this method. However, non-integrative viruses, such as Sendai virus, and non-viral reprogramming systems have emerged as safer alternatives for clinical application (Engel and Ardehali, 2018b; Miyamoto et al., 2018; Tani et al., 2018; Chang et al., 2019).

## Conclusion

As previously discussed, several lncRNAs are expressed during the development of the heart and during heart pathologies. Targeting lncRNAs may be a novel strategy against heart diseases (Bar et al., 2016). Technically, the development of specific and deliverable antisense transcripts (e.g., LNA-GapmeRs) has been proved powerful and efficient carriers for *in vivo* targeting and RNase H-mediated degradation of specific targets (Bernardes de Jesus et al., 2018). Similar approaches may be designed for expression of selected lncRNAs, downregulated in cardiac diseases. We have to face, however, that most human lncRNAs are non-conserved between species, making it extremely challenging to identify the functional lncRNAs *in vivo*. The lack of sequence conservation poses a challenge for the translational application of human lncRNAs. Since lncRNAs are species-specific, we often can only visualize their impact when studied in their specific system. This challenge may only be addressed through a humanized experimental model where the detailed function of non-conserved lncRNAs may be tested. In conclusion, understand lncRNAs specific profiles in dividing vs. non-dividing cardiomyocytes may allow the detection of potentially druggable targets for adult heart repair.

## Author Contributions

FS, MC, SN-P, and BB planned, wrote, and discussed the paper. SN-P and BB revised the paper. All authors contributed to the article and approved the submitted version.

### Conflict of Interest

The authors declare that the research was conducted in the absence of any commercial or financial relationships that could be construed as a potential conflict of interest.

## Supplementary Material

Supplementary Figure 1 Studies on neonatal heart regeneration depicting different methodologies. The studies with blue background observed heart regeneration whether the studies in red could not detect heart healing.
