# Supplementary material for: Age-Related Pathways in Cardiac Regeneration: A Role for lncRNAs?
Source: Front Physiol. 2021 Jan 20;11:583191. doi: 10.3389/fphys.2020.583191 (PMC7855957; doi:10.3389/fphys.2020.583191)
Supplement: Supplementary Figure 1 — Studies on neonatal heart regeneration depicting different methodologies. The studies with blue background observed heart regeneration whether the studies in red could not detect heart healing. [file Image_1.pdf]

Figure 1 - Santos *et al.*

APICAL RESECTION

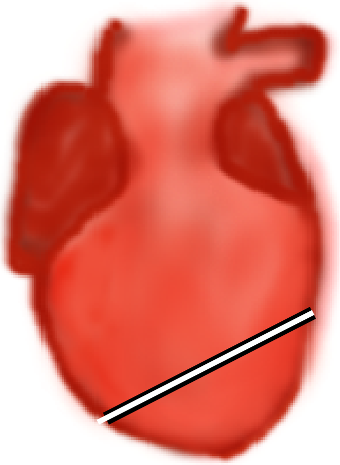

Study	Strain Background	Year
Porrello, E. R.,*et al*	ICR (CD-1)	2011
Mahmoud, A. I.,*et al*	ICR (CD-1)	2014
Sadek, H. A.,*et al*	C57BL/6	2014
Bryant, D. M.,*et al*	ICR (CD-1)	2015
Konfino, T.,*et al*	ICR (CD-1)	2015
Jiang, J.,*et al*	129SvEv	2015
Mahmoud, A. I.,*et al*	ICR (CD-1)	2015
Han, C.,*et al*	ICR (CD-1), C57BL/6J and others	2015
Yu, W.,*et al*	mixed background	2016
Kang, J.,*et al*	ICR (CD-1)	2016
Xiong, J.,*et al*	C57BL/6	2016
Valiente-Alandi, I.,*et al*	mixed background (C57BL/6x129)	2016
Tao, G.,*et al*	mixed background	2016
Bassat, E.,*et al*	ICR (CD-1)	2017
Sampaio-Pinto, V.,*et al*	C57BL/6	2018
Ingason, A. B.,*et al*	ICR (CD-1)	2018
Ahmed, A.,*et al*	mixed background	2018
Elhelaly, W. M.,*et al*	mixed background	2019
Li, Y.,*et al* (b)	several backgrounds	2020
Li, Y.,*et al* (c)	C57BL/6 and B6.129S2	2020
Fan, Y.,*et al*	ICR (CD-1)	2020
Andersen, D. C.,*et al*	C57BL/6	2014
Andersen, D. C.,*et al*	C57BL/6	2016
Zebrowski, D. C.,*et al*	ICR (CD-1)	2017
Cai, W.,*et al*	C57BL/6J	2019

CRYO-INJURY

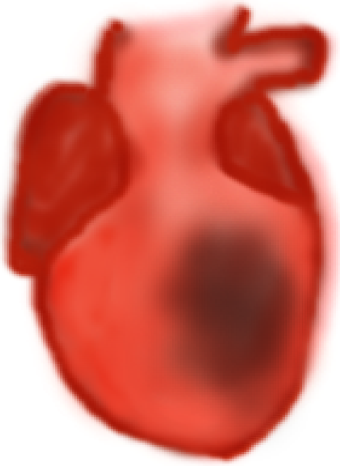

Study	Strain Background	Year
Jesty, S. J.,*et al*	B6.DBA2(F2) or C57BL/6J	2012
Aix, E.,*et al*	C57BL/6	2016
Mohammadi, M. M.,*et al*	SV129/CD-1	2017
Rubin, N.,*et al*	FVB/n	2013
Darehzereshki, A.,*et al*	ICR (CD-1)	2015

LAD LIGATION

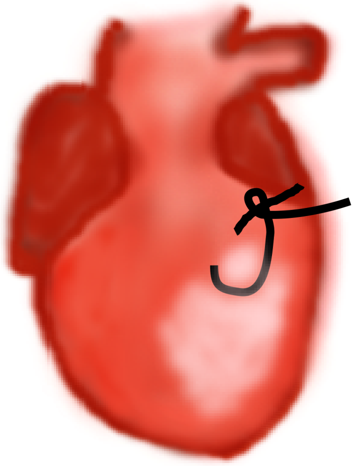

Study	Strain Background	Year
Haubner, B. J.,*et al*	C57BL/6J	2012
Porrello, E. R.,*et al*	ICR (CD-1)	2013
Mahmoud, A. I.,*et al*	C57BL/6xSV129/CD1	2013
Mahmoud, A. I.,*et al*	ICR (CD-1)	2014
Aurora, A. B.,*et al*	ICR (CD-1)	2014
Mahmoud, A. I.,*et al*	ICR (CD-1)	2015
Blom, J. N.,*et al*	C57BL/6x CD-1 IG-S	2016
Haubner, B. J.,*et al*	C57BL/6J, ICR and C57BL/6JxSv129	2016
Ai, S.,*et al*	C57BL/6xSV129	2017
Sereti K-I.,*et al*	mixed background, C57BL/6	2018
Ahmed, A.,*et al*	mixed background	2018
Wang, Z.,*et al*	ICR (CD-1)	2019
Pei, J.,*et al*	C57BL/6	2020
Konfina, T.,*et al*	ICR (CD-1)	2015
